# Genes associated with spontaneous brain activity changes in clinically different patients with major depressive disorder: A transcription‐neuroimaging association study

**DOI:** 10.1111/cns.14311

**Published:** 2023-06-13

**Authors:** Wenshuang Zhu, Feng Liu, Jilian Fu, Wen Qin, Kaizhong Xue, Jie Tang, Yong Zhang, Chunshui Yu

**Affiliations:** ^1^ Department of Radiology and Tianjin Key Laboratory of Functional Imaging Tianjin Medical University General Hospital Tianjin China; ^2^ Tianjin Anding Hospital Tianjin China; ^3^ CAS Center for Excellence in Brain Science and Intelligence Technology, Chinese Academy of Sciences Shanghai China

**Keywords:** amplitude of low‐frequency fluctuations (ALFF), enrichment analysis, gene expression, major depressive disorder (MDD), transcription‐neuroimaging association analysis

## Abstract

**Aims:**

The amplitude of low‐frequency fluctuations (ALFF) of resting‐state functional MRI signals is a reliable neuroimaging measure of spontaneous brain activity. Inconsistent ALFF alterations have been reported in major depressive disorder (MDD) possibly due to clinical heterogeneity. This study was designed to investigate clinically sensitive and insensitive genes associated with ALFF alterations in MDD and the potential mechanisms.

**Methods:**

Transcription‐neuroimaging association analyses of case–control ALFF differences from two independent neuroimaging datasets with gene expression data from Allen Human Brain Atlas were performed to identify the two gene sets. Various enrichment analyses were conducted to characterize their preference in biological functions, cell types, temporal stages, and shared effects with other psychiatric disorders.

**Results:**

Compared with controls, first‐episode and drug‐naïve patients showed more extensive ALFF alterations than patients with varied clinical features. We identified 903 clinically sensitive genes and 633 clinically insensitive genes, and the former was enriched for genes with down‐regulated expression in the cerebral cortex of MDD patients. Despite shared functions of cell communication, signaling, and transport, clinically sensitive genes were enriched for cell differentiation and development whereas clinically insensitive genes were for ion transport and synaptic signaling. Clinically sensitive genes showed enrichment for microglia and macrophage from childhood to young adulthood in contrast to clinically insensitive genes for neurons before early infancy. Clinically sensitive genes (15.2%) were less likely correlated with ALFF alterations in schizophrenia than clinically insensitive genes (66.8%), and both were not relevant to bipolar disorder and adult attention deficit and hyperactivity disorder based on a third independent neuroimaging dataset.

**Conclusions:**

Present results provide novel insights into the molecular mechanisms of spontaneous brain activity changes in clinically different patients with MDD.

## Graphical abstract

Clinically sensitive and insensitive genes associated with alterations of the amplitude of low‐frequency fluctuations (ALFF) in major depressive disorder (MDD) are identified. The two gene sets are differentially enriched for cortical differential expression genes in MDD, biological functions, developmental periods, cell types, and genes associated with ALFF alterations in SCZ.

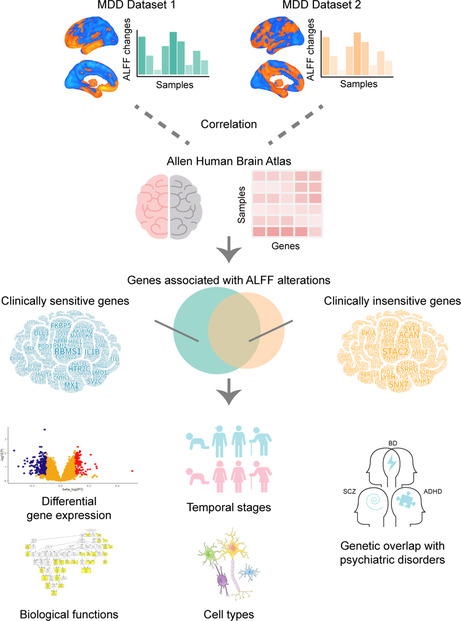

## 1 INTRODUCTION

Major depressive disorder (MDD) is a common and complex psychiatric disorder characterized by continuous loss of interest, depressed mood, and decreased energy. This disorder affects more than 300 million people in the world and ranks as the leading cause of global disability.^1^ Although the etiologies of MDD are poorly understood, current evidence supports that both genetic and environmental factors are associated with MDD.^2^ In twin studies,^3^, ^4^ the estimated heritability of MDD is around 37%–40%, and genome‐wide association studies (GWASs) have identified more than a hundred of risk genetic loci for this disorder.^5^

In addition to task‐induced activation, the functional magnetic resonance imaging (fMRI) can also measure spontaneous brain activity. For instance, the amplitude of low‐frequency fluctuations (ALFF) of blood–oxygen‐level dependent signals can measure spontaneous brain activity with high test–retest reliability,^6^, ^7^ which is the foundation for the calculation of functional connectivity and functional brain networks. ALFF alterations in multiple brain regions have been reported in many studies on MDD.^8^, ^9^, ^10^ However, the reported ALFF alterations in MDD were inconsistent, which may be related to clinical differences across studies, such as the number of episodes, disease durations, and anti‐depressive medications. Nevertheless, the genetic bases underlying the heterogeneous case–control ALFF differences between clinically different patients with MDD remain unclear.

Several candidate gene studies^11^, ^12^, ^13^ have reported interactions between genetic variants and MDD diagnosis on ALFF phenotypes, indicating the genetic regulation of ALFF alterations in MDD. However, the candidate gene approach can only test for the associations between a single or a few genetic variants and a phenotype, increasing the risk of selective reporting and false positives. The drawback can be overcome by GWAS, which can simultaneously interrogate millions of variants to unbiasedly identify the associations between genetic variants with the phenotype. In terms of spontaneous brain activity, GWASs have identified genome‐wide significant associations of amplitudes of resting‐state networks derived from resting‐state fMRI data.^14^ However, the GWAS approach generally requires a large sample size to obtain positive findings, cannot be used to investigate tissue‐specific associations since genetic variants are similar across tissues and to identify the associations between genetic variants and case–control ALFF differences of MDD, and the GWAS results are difficult to be interpreted because most GWAS‐identified genetic variants locate in intergenic regions with unknown functions and are seldom the causal variants. In contrast, transcription‐neuroimaging association analyses can overcome these GWAS drawbacks, which has been used to identify genes associated with imaging phenotypes in the brain,^15^, ^16^ such as the genes associated with fractional ALFF in the default‐mode network.^17^ Moreover, many transcription‐neuroimaging association studies have reported gene expression profiles that are associated with brain structural and functional changes in patients with psychiatric disorders.^18^, ^19^, ^20^, ^21^, ^22^, ^23^, ^24^ However, no studies have been conducted to identify the associations between brain gene expression and ALFF alterations in MDD. By comparing gene sets associated with clinically different neuroimaging datasets of MDD, we can distinguish clinically sensitive and insensitive genes associated with case–control ALFF differences of MDD.

**FIGURE 1:**
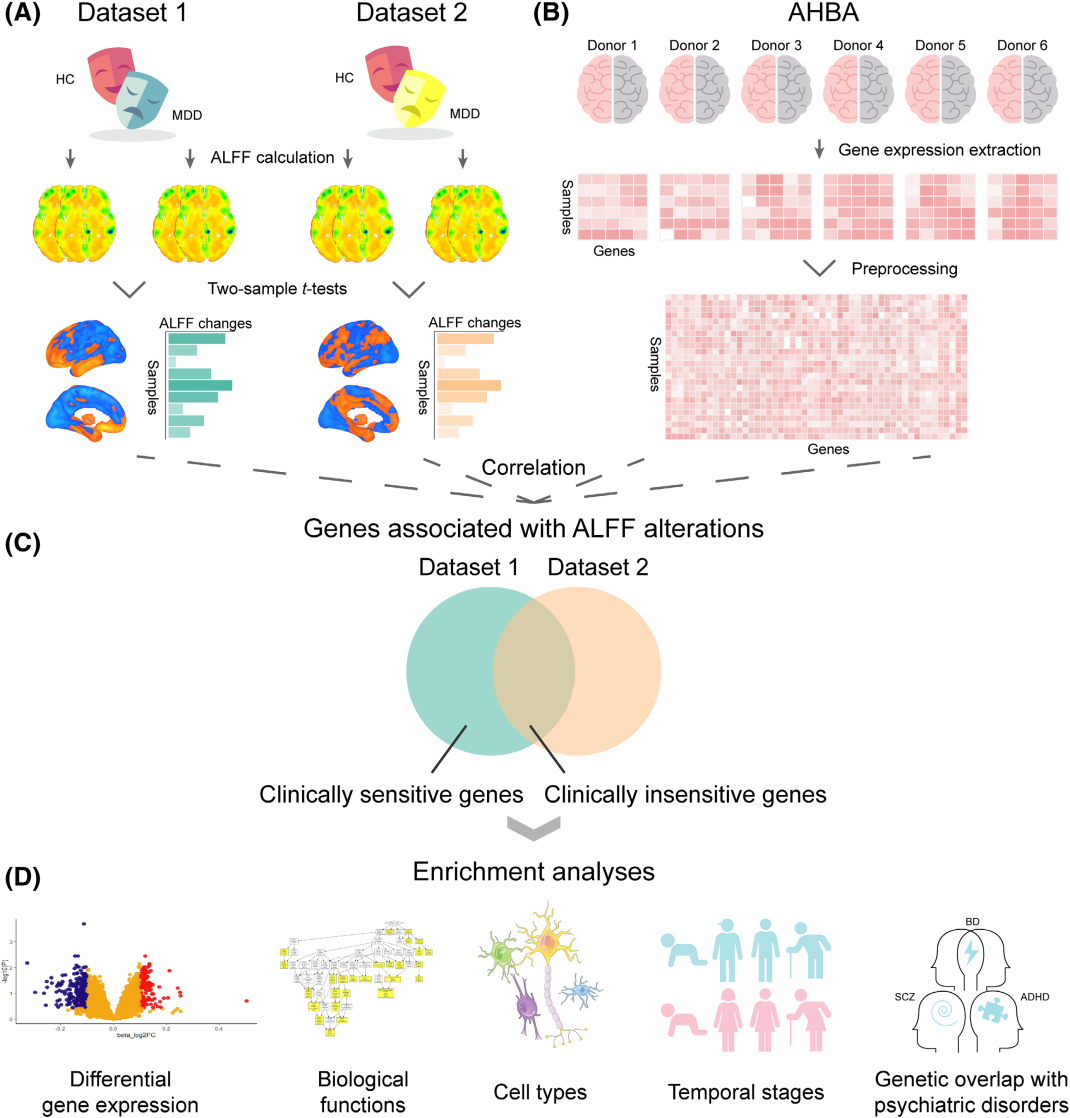
A schematic workflow of the study protocol. (A) Neuroimaging data preprocessing and measure extraction. (B) Transcriptional data preprocessing. (C) Identification of clinically sensitive and insensitive genes associated with ALFF alterations of MDD. (D) Enrichment analyses of gene sets. ADHD, attention deficit hyperactivity disorder; AHBA, Allen human brain atlas; ALFF, amplitude of low‐frequency fluctuations; BD, bipolar disorder; HC, healthy controls; MDD, major depressive disorder; SCZ, schizophrenia.

In this study, the transcriptome‐neuroimaging association analysis was conducted based on gene expression data derived from the Allen Human Brain Atlas (AHBA)^25^ and case–control ALFF differences between patients with MDD and healthy controls. The case–control ALFF differences were derived from two independent datasets with clinically different patients with MDD: dataset 1 included first‐episode and drug‐naïve patients and dataset 2 included patients with varied duration, medication, and episodes. First, we compared voxel‐wise ALFF differences between patients and controls from the two datasets, respectively. Second, we conducted gene‐wise across‐sample spatial correlations between gene expression profiles and ALFF alterations accounting for spatial autocorrelation to identify genes associated with ALFF alterations in dataset 1 and dataset 2, respectively. Based on the results, we distinguished clinically sensitive (genes only significant in dataset 1) and insensitive (genes significant in both datasets) genes. Third, a series of functional annotations were conducted for the two gene sets, respectively. Finally, we investigated the genetic effects on ALFF alterations shared by MDD and other psychiatric disorders including schizophrenia (SCZ), bipolar disorder (BD), and adult attention deficit and hyperactivity disorder (ADHD). A schematic workflow of the study protocol is shown in Figure 1.

## 2 MATERIALS AND METHODS

### 2.1 Subjects

Dataset 1 included 55 first‐episode drug‐naïve patients with MDD and 56 age‐ and sex‐matched healthy controls recruited from January 2017 to June 2018. Dataset 2 included 55 patients with MDD with varied duration, medication, and episodes and 53 age‐ and sex‐matched controls recruited from March 2013 to March 2014. Demographics of these two datasets are provided in Table 1. All patients were diagnosed with MDD according to the structured clinical interview for DSM‐IV (SCID). The severity of depressive symptoms was assessed by the 17‐item Hamilton Depression Rating Scale (HDRS‐17).^26^ Healthy controls were recruited from the local community and were screened by the SCID Non‐Patient Edition. Exclusion criteria for all subjects included a history of loss of consciousness due to head injury, drug abuse or alcohol dependency, presence of pregnancy, or contraindications of MRI examination. Additionally, healthy controls and their first‐degree relatives were required to be free from a history of mental disorders. This study was approved by the ethical committee of the Tianjin Medical University General Hospital, and written informed consent was obtained from each subject.

**TABLE 1:** Demographics of the MDD datasets.

Demographics	Sex (male/female)	Age (years) (mean ± SD)	Mean FD (mm) (mean ± SD)	HDRS‐17 score (mean ± SD)	Duration (months) (mean ± SD)
Dataset 1 (54 MDD vs. 56 HC)					
MDD	19/35	35.4 ± 12.7	0.06 ± 0.04	27.2 ± 5.6	19.9 ± 39.8
HC	24/32	39.0 ± 13.4	0.06 ± 0.05	/	/
Statistics	*χ* ^2^ = 0.680	*z* = −1.478^a^	*z* = −0.622^a^	/	/
*p*‐Values	0.410	0.139	0.534	/	/
Dataset 2 (53 MDD vs. 52 HC)					
MDD	21/32	39.5 ± 13.6	0.05 ± 0.03	27.8 ± 10.6	36.5 ± 62.0
HC	24/28	36.6 ± 10.4	0.05 ± 0.04	/	/
Statistics	*χ* ^2^ = 0.457	*z* = −1.171^a^	*z* = −0.628^a^	/	/
*p*‐Values	0.499	0.242	0.530	/	/

*Note*: Of note, four subjects are excluded because of poor imaging quality. HDRS‐17 scores and durations are assessed for 53 patients from dataset 1 and 45 patients from dataset 2, respectively.

Abbreviations: FD, framewise displacement; HC, healthy controls; HDRS‐17, Hamilton Depression Rating Scale; MDD, major depressive disorder; SD, standard deviation.

^a^ Mann–Whitney *U*‐test. Normality is tested using the Lilliefors test.

### 2.2 MRI data acquisition and preprocessing

All MRI data were acquired by a 3.0‐Tesla MR scanner (Discovery MR750, General Electric). All participants were asked to stay awake, keep their eyes closed, relax, and think of nothing during the scans. Earplugs and foam paddings were used to reduce scanner noise and minimize head motion. Resting‐state fMRI data were acquired using a gradient‐echo single‐shot echo‐planar imaging sequence and Sagittal 3D T1‐weighted MRI data using a brain volume sequence. For the detailed acquisition parameters, please see Table S1.

The fMRI data preprocessing was performed using the Data Processing Assistant for Resting‐State fMRI (DPARSFA version 4.3, http://rfmri.org/DPARSF).^27^ Briefly, the first 10 volumes were removed due to signal instability. For the remaining 170 volumes, slice timing was conducted by realigning them to the first volume to correct for acquisition delay and head motion. Four subjects (one patient in dataset 1, and two patients and one control in dataset 2) with head motion exceeding translational 2 mm or rotational 2° were excluded. We also calculated mean framewise displacement (FD) to index volume‐to‐volume movement,^28^ and no significant differences in FD were found between the two datasets. For spatial normalization, the structural images were linearly registered to the realigned mean functional image and segmented into gray matter, white matter, and cerebrospinal fluid. Next, the segmented tissue‐specific images were normalized to the standard Montreal Neurological Institute (MNI) space using the non‐linear diffeomorphic anatomical registration through exponentiated Lie algebra technique,^29^ and the estimated deformation parameters were utilized to transform the functional images from individual space to standard MNI space. Subsequently, the normalized functional images were resampled to 3‐mm voxel size and smoothed with an 8‐mm full‐width at half‐maximum Gaussian Kernel. Finally, nuisance variables including linear trend, white matter signal, cerebrospinal fluid signal, and Friston‐24 head motion parameters^30^ were regressed out.

### 2.3 ALFF calculation and intergroup comparison

The ALFF maps were calculated via DPARSFA by the following two steps. First, the time series of each voxel were fast Fourier‐transformed into the frequency domain to acquire the power spectrum, and the square root of the power spectrum was computed and averaged in the frequency range of 0.01–0.08 Hz to obtain the raw ALFF map. Second, the ALFF value of each voxel was divided by the global mean ALFF value for standardization. Due to the ALFF values of nearly a half of voxels without the normal distribution in the Lilliefors test, non‐parametric permutation test (5000 times) was used to test ALFF differences between patients with MDD and healthy controls, while controlling for age, sex, and mean FD. Multiple comparisons were corrected using the voxel‐level family‐wise error (FWE) of *p* < 0.05 (Figure 1A). We also calculated the case–control ALFF difference maps using other statistical thresholds (*p* < 0.001, *p* < 0.01, *p* < 0.05, and unthresholded) and overlapping voxels with consistent directions of effects between the two datasets to show the consistency and difference between the two ALFF alteration maps of MDD.

The spatial correlation between ALFF *t*‐maps derived from the two datasets was conducted by voxel‐wise Pearson's correlation. The significance was assessed by 1000 permutation tests to eliminate the autocorrelation effects in neuroimaging maps. Specifically, for each *t*‐map, 1000 surrogate brain maps that preserved the original autocorrelation properties to represent the randomized phenotypes were created by BrainSMASH (Brain Surrogate Maps with Autocorrelated Spatial Heterogeneity),^31^ and Pearson's correlation was repeated between the real map and surrogate maps. Then *p*‐value was obtained based on the location of the true correlation coefficient in the null distribution.

### 2.4 Gene expression data

The brain‐wide gene expression data were obtained from the publicly available AHBA database, which provides comprehensive and high‐resolution coverage of normalized microarray expression data in six donated post‐mortem brains (Table S2), including 3702 samples measured by 58,692 probes.^25^ Of note, two donors had expression data in both hemispheres and the other four donors had expression data only in the left hemisphere. According to the proposed analytic pipeline,^32^ the expression data were processed by the following steps: (i) all of these 58,692 probes were re‐assigned to genes by Re‐Annotator toolkit^33^ using the genome assembly hg38 in line with the latest database at the National Center for Biotechnology Information, and 47,795 probes corresponding to 20,919 unique genes were identified after reannotation; (ii) probes exceeding the background noise in at least 50% of samples were retained in the intensity‐based filtering step; (iii) since AHBA offered the RNA‐seq datasets of two donors, probes showing correlation coefficients greater than 0.2 with RNA‐seq measurements were retained and the probe showing the highest correlation with RNA‐seq data was selected to represent the gene expression level; (iv) the samples of the left cerebral cortex which were mapped to Desikan‐Killany atlas^34^ within a threshold of 2 mm Euclidean distance were kept because all donors had the left hemisphere's data; (v) scaled robust sigmoid normalization was performed to minimizing the gene expression differences between donors and between brain regions; (vi) differential stability (DS) score was calculated to evaluate consistency of gene expression patterns across individuals.^35^ Finally, an expression matrix of 10,185 × 1285 (i.e., genes × samples) was obtained for the subsequent analyses (Figure 1B).

### 2.5 Transcriptome‐neuroimaging association analysis

Based on the unthresholded voxel‐wise *t*‐map representing ALFF differences between patients with MDD and healthy controls, the mean *t*‐value in the 6 mm radius sphere centered at the MNI coordinate of each tissue sample was defined as the ALFF difference of this sample. Then cross‐sample correlations between ALFF differences and transcriptional profiles were performed by Pearson's correlation to identify genes whose expression levels were correlated with ALFF alterations in MDD. Considering the autocorrelation effects in transcriptional and neuroimaging maps, permutation tests (1000 times) were performed in each dataset to measure how likely this result would be obtained by chance. Specifically, in each permutation, the same transcriptome‐neuroimaging association analysis was performed using the above‐mentioned surrogate brain maps for each ALFF difference map from BrainSMASH, and the maximum absolute correlation coefficient (i.e., *r*‐value) in all genes was recorded. Based on the location of the true correlation coefficient of each gene in the constructed null distribution of 1000 maximal absolute correlation coefficients, we can infer the statistical significance of this gene (*p*_*perm*_ < 0.05, FWE corrected). Finally, the significant genes with DS value ranked in the top 50% were considered as genes associated with ALFF alterations in MDD (Figure 1C). The transcriptome‐neuroimaging association analysis was conducted for the case–control ALFF difference map from dataset 1 and dataset 2, respectively. Based on the results, we distinguished clinically sensitive (genes only significant in dataset 1) and insensitive (genes significant in both datasets) genes.

### 2.6 Enrichment for genes with differential expression in MDD

Enrichment analysis was conducted to determine the relation between genes (clinically sensitive and insensitive genes) identified by this study and those with differential gene expression in the cerebral cortex in MDD patients.^36^ Fisher's exact test (*p* < 0.05) was used to examine the significance (Figure 1D), in which the background genes were the re‐annotated 20,919 brain‐expressed genes of the whole genome (the background genes were applied for all enrichment analyses).

### 2.7 Enrichment for biological functions

To explore the biological functions of the identified genes, functional annotations of clinically sensitive and insensitive genes were carried out by the biological process of gene ontology (GO) implemented in the online tool *g:Profiler*.^37^ Besides, considering the false‐positive bias caused by within‐category gene–gene co‐expression and spatial autocorrelation in the enrichment analysis based on the spatial transcriptomic data,^38^ ensemble‐based null models were applied to reperform the functional enrichment and test the reliability of the reported annotations. In this model, available GO term hierarchy and annotation files were derived from the enrichment toolbox (https://github.com/benfulcher/GeneSetEnrichmentAnalysis, data version: April 17, 2019). For each GO category, the category score was defined as the average of the absolute values of Pearson's correlation coefficients obtained in the transcriptome‐neuroimaging association analysis for genes in this category. The null model of category‐level scores was constructed by the same procedure based on the ensemble of 1000 randomized phenotypes while preserving spatial autocorrelation. By comparing the category score to a null distribution of category‐level scores, we performed the statistical inference on GO category and tested whether the ALFF alterations were more correlated to genes in this category than random phenotypes. The analysis was conducted separately for the clinically sensitive and insensitive genes. All the tests were Bonferroni corrected (*p* < 0.05).

### 2.8 Temporal‐specific expression analysis

Since gene expression is dynamic over time, the specificity index threshold (pSI) package^39^ was used to investigate the specific expression stage of the two gene sets during the lifetime. In this package, the pSI of each gene in each developing stage was calculated based on transcriptome profiles derived from the BrainSpan database (http://www.brainspan.org), which indicates the possibility of specific expression of a gene in each developmental stage relative to others. Significant periods were obtained by Fisher's exact test at pSI value of 0.01, followed by Bonferroni correction for the numbers of gene sets and stages (*p* < 0.05). In addition, the human brain transcriptome database was utilized to show the representative gene expression patterns.^40^

### 2.9 Cell type‐specific expression analysis

Since the brain consists of various types of cells with specific gene expression, cell type‐specific analysis was performed to explore the specific cell types associated with the two gene sets. Cell type‐specific gene expression data of the human brain neurons, astrocytes, oligodendrocytes, microglia, and macrophage, were downloaded from the CellMarker database.^41^ Fisher's exact test was used to identify in which cell type each gene set was overexpressed, followed by Bonferroni correction for the numbers of gene sets and cell types (*p* < 0.05).

### 2.10 Genetic overlaps with ALFF alterations of other major psychiatric disorders

MDD has been found to share genetic architecture with other major psychiatric diseases.^36^ To explore whether genes associated with ALFF alterations in MDD are also associated with ALFF changes in other major psychiatric disorders, we performed the gene‐neuroimaging association analysis using a public fMRI resource of SCZ (29 patients), BD (36 patients), ADHD (33 patients), and matched healthy controls (28 for SCZ, 34 for BD and 32 for ADHD).^42^ The fMRI and T1‐weighted images were obtained from the OpenfMRI database (https://openfmri.org/dataset/ds000030/). The accession number of this study was ds000030. Subjects with imaging artifacts and head motion parameters exceeding translational 2 mm or rotational 2° were excluded. The preprocessing of these neuroimaging data and transcriptome‐neuroimaging association analysis of each disorder was the same as MDD. Details of demographics and scan parameters are shown in Tables S3 and S4.

## 3 RESULTS

### 3.1 ALFF differences between patients with MDD and healthy controls

**FIGURE 2:**
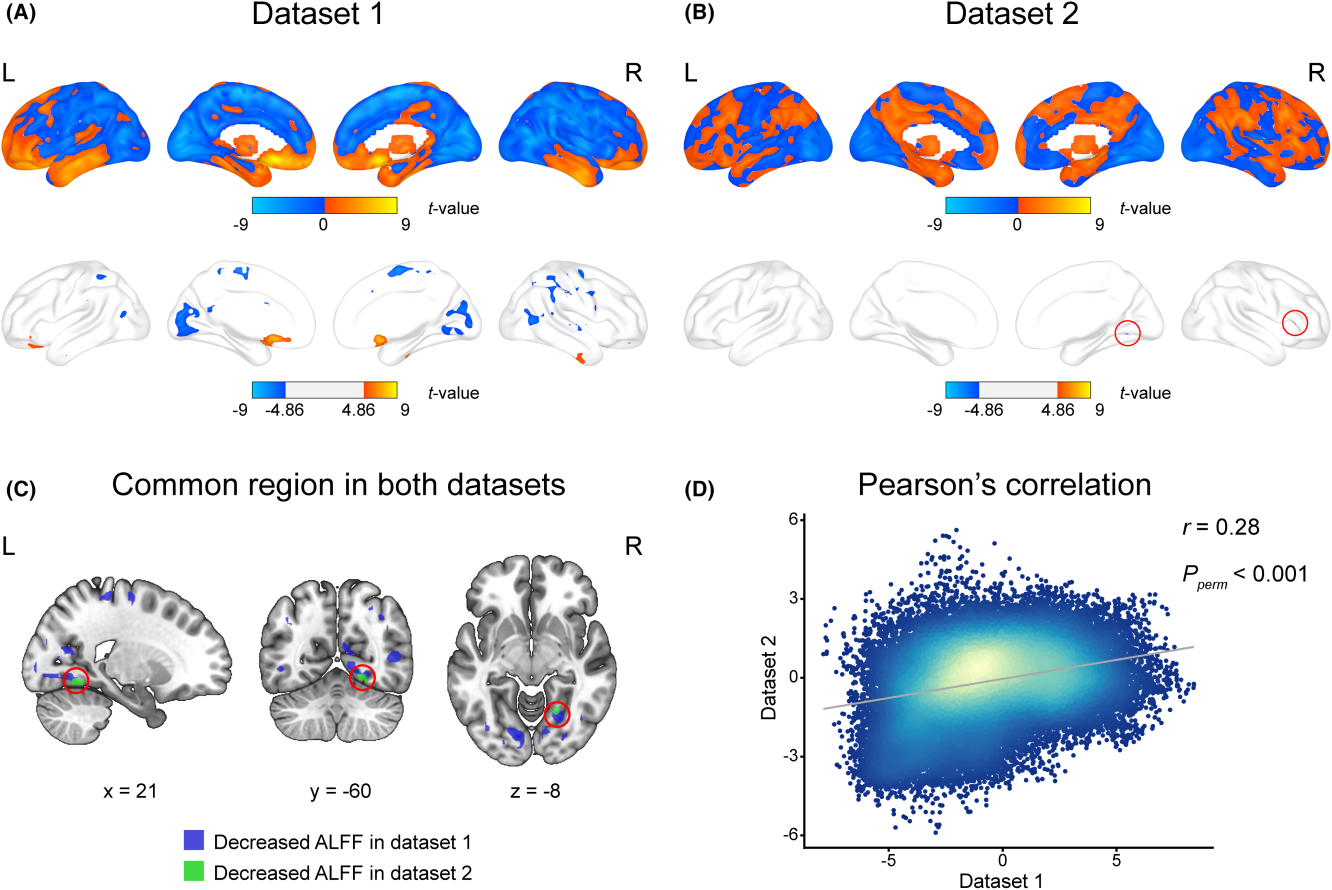
ALFF alterations between patients with MDD and healthy controls. (A) The case–control ALFF differences in dataset 1 without (upper row) and with (lower row) a cutoff threshold (voxel‐level FWE correction *p* < 0.05). (B) The case–control ALFF differences in dataset 2 without (upper row) and with (lower row) a cutoff threshold. The red circles show the significantly altered ALFF regions. (C) Common regions (red circle) with significantly decreased ALFF differences in both datasets (voxel‐level FWE correction *p* < 0.05). (D) Voxel‐wise Pearson's correlation between ALFF difference maps without cutoff threshold from the two datasets. L, left; R, right.

In dataset 1 with the strictest statistical threshold (*p* < 0.05, FWE corrected), we found that MDD patients showed increased ALFF mainly in the frontal and temporal lobes, especially the bilateral inferior frontal gyri, and decreased ALFF mainly in the parietal and occipital lobes, such as the bilateral calcarine, cuneus and lingual gyri (Figure 2A; Table S5) compared with healthy controls. In dataset 2, we observed increased ALFF in the right inferior frontal gyrus and decreased ALFF in the right lingual and fusiform gyrus (Figure 2B; Table S5). Although the right fusiform gyrus showed decreased ALFF in both datasets (Figure 2C), the ALFF alterations (*p* < 0.05, FWE corrected) were more extensive in dataset 1 than in dataset 2. However, in the unthresholded statistical maps of the two datasets, 23,350/40,857 (57.15%) voxels showed consistent directions of effects between datasets (Figure 2A,B). Moreover, the overlapping voxels between the two datasets were increased with the relaxation of statistical thresholds (Figure S1; Table S6). The voxel‐wise spatial correlation analysis also showed a significant positive correlation (*r* = 0.28, *p*_*perm*_ < 0.001, Figure 2D) between the *t*‐maps of case–control ALFF differences of the two datasets.

### 3.2 Associations between gene expression profiles and ALFF alterations of MDD

**FIGURE 3:**
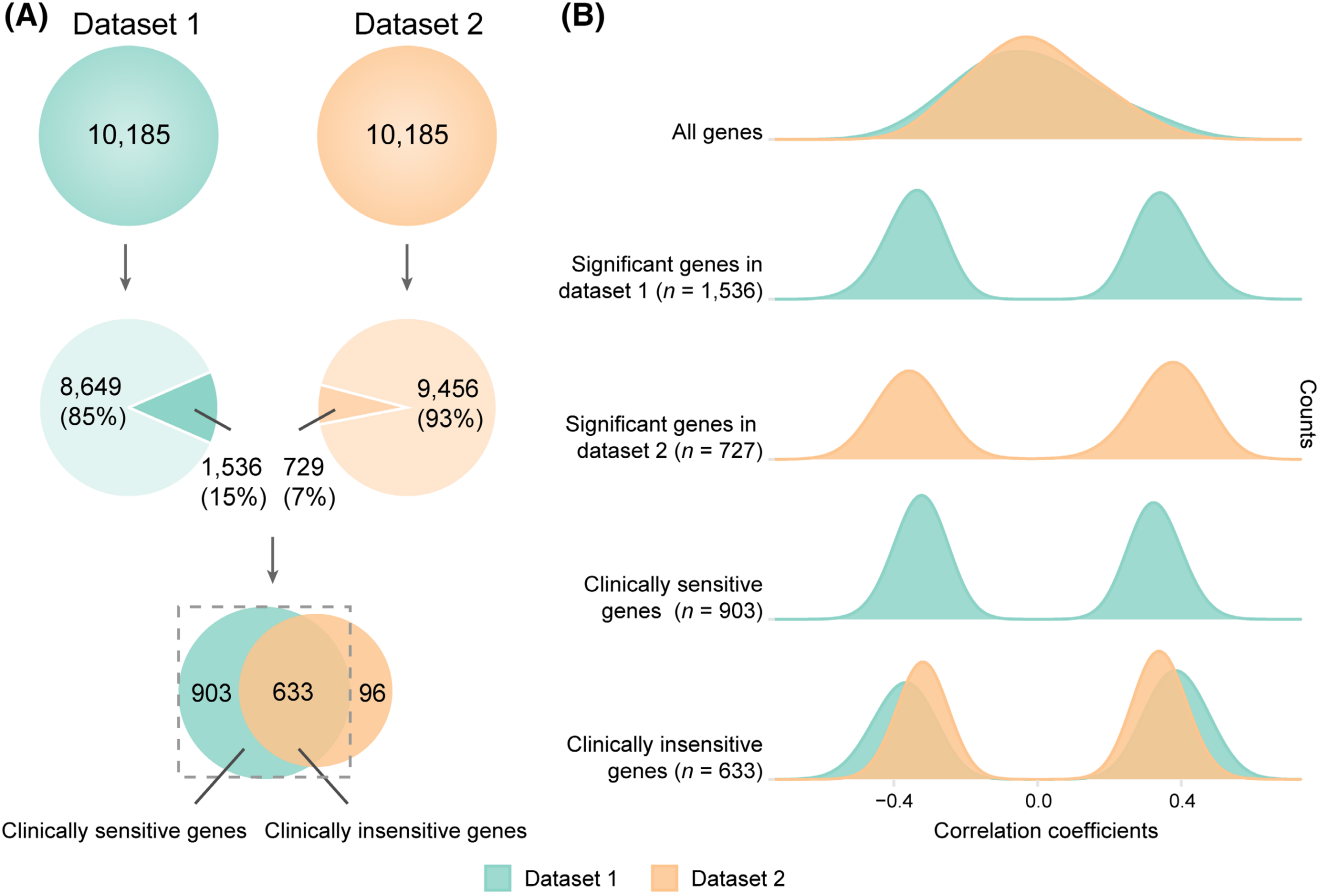
Discovered genes in transcriptome‐neuroimaging association analyses and distributions of their correlation coefficients. (A) Discovered genes in transcriptome‐neuroimaging association analyses. A total of 10,185 genes are included in transcriptome‐neuroimaging association analyses for both datasets. 1536 genes are correlated with ALFF alterations in MDD in dataset 1, of which 903 genes are not replicated and 633 genes are replicated in dataset 2. The 903 genes are defined as clinically sensitive genes and the 633 genes are defined as clinically insensitive genes. (B) The distributions of correlation coefficients of gene expression with ALFF alterations in MDD. ALFF, amplitude of low‐frequency fluctuations; MDD, major depressive disorder.

Gene‐wise across‐sample spatial correlations between gene expression and case–control ALFF difference were performed separately for each neuroimaging dataset (Figure 3A). With a threshold of Bonferroni and permutation corrected *p* < 0.05 and DS > 50% (DS = 0.2433), 1536 genes were spatially correlated with case–control ALFF difference in dataset 1 and 729 genes in dataset 2, of which 633 genes showed consistent spatial correlations (the same statistical threshold and effect direction) with case–control ALFF difference in both datasets (Figure 3B; Table S7). The 903 genes identified only in dataset 1 were defined as the clinically sensitive gene set because the associations between gene expression of these genes and case–control ALFF difference in MDD are sensitive to clinical conditions, such as duration, medication, and episodes. The 633 genes shared by the two datasets were defined as the clinically insensitive gene set because the associations between gene expression of these genes and case–control ALFF difference in MDD are insensitive to clinical conditions. These two gene sets were included in the following enrichment analyses.

### 3.3 Enrichment for genes with differential expression in MDD

Differential gene expression data in the cerebral cortex of MDD patients were used for enrichment analysis of the two gene sets. We found that the 903 clinically sensitive genes showed significant enrichment for down‐regulated genes in the post‐mortem cortex of MDD patients (*p* = 3.32 × 10^−4^), but not for up‐regulated genes (*p* = 0.9383). The 633 clinically insensitive genes were not enriched for either up‐regulated (*p* = 1) or down‐regulated genes (*p* = 0.4408) (Table 2).

**TABLE 2:** Enrichment for genes with differential expression in MDD.

Gene category	Odds ratios	*p*‐Values	Overlapped genes
Clinically sensitive genes (*n* = 903)			
Up‐regulated genes	0.44	0.94	2/183
Down‐regulated genes	3.20	3.32 × 10^−4^	14/145
Clinically insensitive genes (*n* = 633)			
Up‐regulated genes	0	1	0/183
Down‐regulated genes	1.19	0.44	4/145

### 3.4 Enrichment for biological functions

**FIGURE 4:**
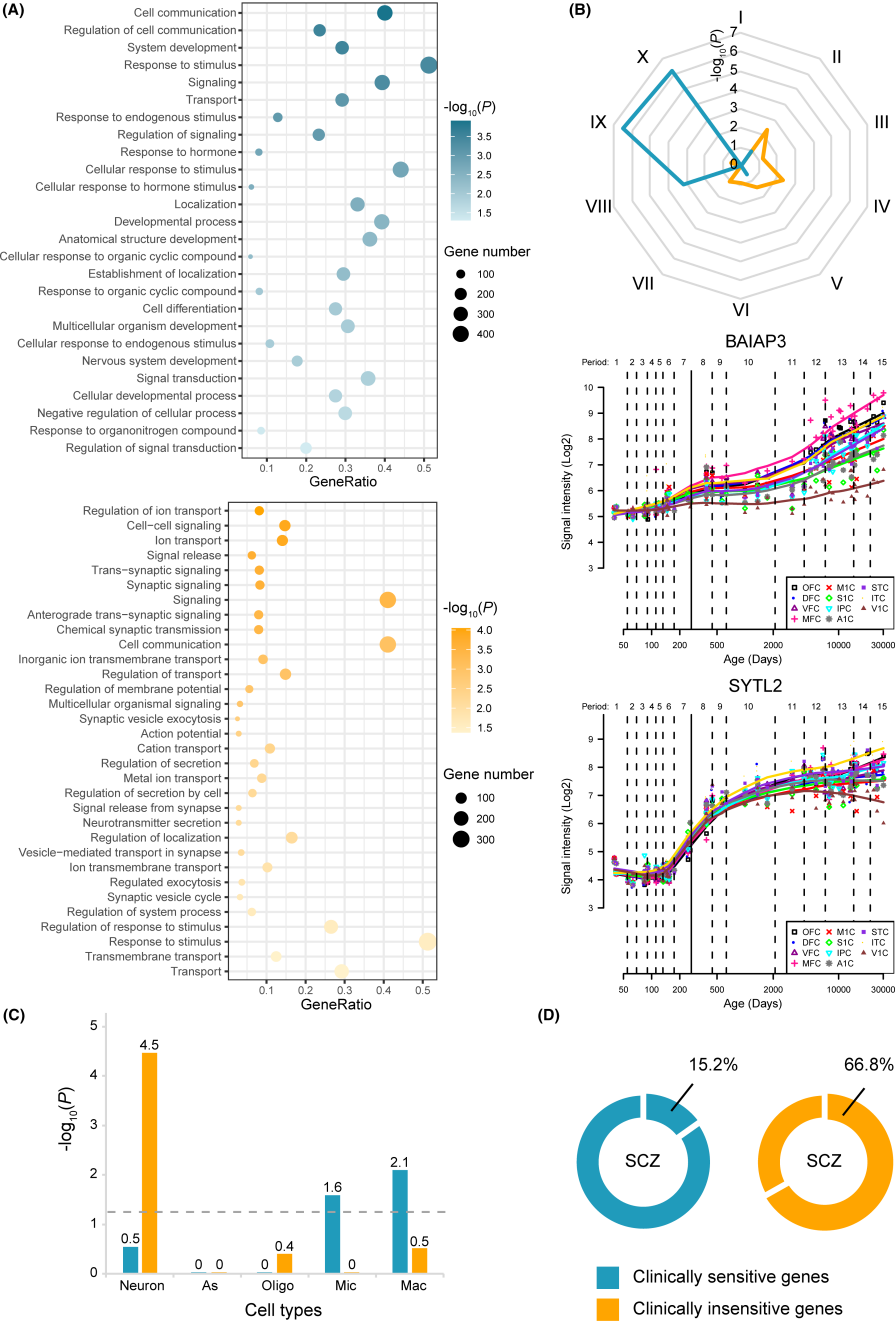
Enrichment analyses of the two gene sets. (A) Enrichments of clinically sensitive (upper panel) and insensitive (lower panel) genes for GO items of biological processes. Only items passed the Bonferroni‐corrected threshold are displayed. The x‐axis shows the gene ratio (intersection size/query size) of each item, the y‐axis shows GO terms, the bubble size indicates the intersecting number of genes in the category, and the colors of bubbles represent significance. (B) Temporal‐specific enrichment analysis and expression patterns of representing genes. Each Roman number represents a temporal stage: I, early fetal; II, early mid fetal; III, late mid fetal; IV, late fetal; V, neonatal and early infancy; VI, late infancy; VII, early childhood; VIII, middle and late childhood; IX, adolescence; X, young adulthood. *BAIAP3* is a representative gene from the clinically sensitive gene set, which shows high expression at the late developmental stages. *SYTL2* is a representative gene from the clinically insensitive gene set, which shows a rapid increase in expression at the early developmental stages. (C) Cell type‐specific analysis. The x‐axis shows the cell type, the y‐axis shows the −log_10_(*p*) value, and the gray dashed line indicates the threshold of significance. (D) The proportion of the two sets of genes associated with ALFF alterations in SCZ. A1C, primary auditory cortex; DFC, dorsolateral prefrontal cortex; GO, Gene Ontology; IPC, posterior inferior parietal cortex; ITC, inferior temporal cortex; M1C, primary motor cortex; MFC, medial prefrontal cortex; OFC, orbital prefrontal cortex; S1C, primary somatosensory cortex; STC, superior temporal cortex; V1C, primary visual cortex; VFC, ventrolateral prefrontal cortex.

GO enrichment analyses were conducted to find neurobiologically relevant biological functions of the two gene sets. Although both clinically sensitive and insensitive genes were enriched for cell communication, response to stimulus, signaling, and transport (Figure 4A), they also showed relatively specific enrichments. For example, clinically sensitive genes were preferentially enriched for cell development and differentiation, and clinically insensitive genes were involved in ion transport and synaptic signaling. All the reported biological processes were still significant when using the ensemble‐based null models with Bonferroni correction. The full lists of enriched terms and corresponding *p‐*values are presented in Tables S8 and S9.

### 3.5 Temporal‐specific expression enrichment

In the temporal‐specific expression analysis, the clinically sensitive genes showed overexpression in the relatively late developmental stages, including the middle and late childhood (*p* = 6.46 × 10^−4^), adolescence (*p* = 2.87 × 10^−7^), and young adulthood (*p* = 6.58 × 10^−7^); however, the clinically insensitive genes were specifically expressed in the relatively early developmental stages, including the early/mid fetal (*p* = 0.0047), late fetal (*p* = 0.0045), and neonatal and early infancy (*p* = 0.0039). The different expression patterns of the representing genes were characterized based on the Human Brain Transcriptome database (Figure 4B).

### 3.6 Cell type‐specific enrichment

Cell type‐specific expression analysis identified the specific overexpression of the two gene sets in three given human brain cell types. Specifically, the clinically sensitive genes were significantly enriched for microglia (*p* = 0.0261) and macrophage (*p* = 0.0079), and the clinically insensitive genes were significantly enriched for neurons (*p* = 3.43 × 10^−5^) (Figure 4C).

### 3.7 Genetic overlaps with ALFF alterations of other major psychiatric disorders

Taking ALFF alteration as an intermediate phenotype, we assessed the shared genetic components of MDD with other psychiatric disorders at the transcriptome level by transcriptome‐neuroimaging association analyses. The ALFF alterations in SCZ, BD, and ADHD are shown in Figure S2. With the same correction threshold, 616 genes, 17 genes, and 1 gene were associated with ALFF alterations in SCZ, BD, and ADHD, respectively (Table S10). Taken together, 15.17% (137/903) clinically sensitive genes were significantly correlated with ALFF alterations in SCZ, and 66.82% (423/633) clinically insensitive genes were correlated with ALFF alterations in SCZ. However, none of the identified genes were correlated with ALFF alterations in BD and ADHD (Figure 4D).

## 4 DISCUSSION

In the present study, we performed transcriptome‐neuroimaging association analyses to identify gene expression profiles associated with ALFF alterations in MDD in two independent neuroimaging datasets with different clinical features (first‐episode drug‐naïve patients vs. patients with varied duration, medication, and episodes). From which, we distinguished 903 clinically sensitive genes (significant in dataset 1 only) and 633 clinically insensitive genes (significant in both datasets). Clinically sensitive and insensitive genes differed in enrichments for cortical differential expression genes in MDD, biological functions, developmental periods, cell types, and genes associated with ALFF alterations in SCZ, although they shared function annotations such as cell communication, response to stimulus, signaling, and transport. These results may shed light on the molecular mechanisms underlying ALFF alterations in MDD and provide potential biological explanations for the inconsistent ALFF alterations reported in MDD patients with different clinical features.

Under the strictest statistical threshold, we found ALFF reduction in the right fusiform gyrus in both neuroimaging datasets, and the right fusiform gyrus also showed abnormal activation in facial task^43^, ^44^ and reduced cortical thickness and volume in MDD.^45^ Since ALFF reflects spontaneous brain activity, our finding indicates that spontaneous brain activity in the right fusiform gyrus is impaired in MDD, supporting that the structural and functional alterations in the right fusiform gyrus are potential neuroimaging markers of MDD.^46^, ^47^ Compared with extensive ALFF alterations in first‐episode and drug‐naïve patients with MDD, ALFF alterations were restricted in MDD patients with varied duration, medication and episodes, which agrees with the observation that antidepressants can attenuate functional abnormalities in MDD.^48^ Due to great clinical heterogeneity, the variability between the two datasets is expected for the strictest multiple testing correction. However, the coexistence of consistency and difference between datasets indicates that the unthresholded ALFF alteration maps of MDD can provide enough information to find consistent and different transcription‐neuroimaging associations between the two datasets.

Based on the transcriptome‐neuroimaging associations in the two datasets, we categorized genes associated with ALFF alterations in MDD into clinically sensitive and insensitive genes. Clinically sensitive genes were associated with ALFF alterations in first‐episode drug‐naïve patients, but not associated with ALFF alterations in patients with antidepressant treatments and disease progression, which are potential candidates for the investigation of genetic mechanisms underlying the currently used antidepressant treatments. For instance, clinically sensitive genes showed enrichment for genes with down‐regulated expression in the cerebral cortex from patients with MDD, indicating that the current antidepressant treatments may act by affecting at least a portion of these down‐regulated genes. In contrast, clinically insensitive genes were associated with ALFF alterations in MDD irrespective of clinical features including medication, which are useful for the discovery of novel molecular targets for treatments since these genes are insensitive to the currently used treatments.

Enrichment analyses provide further biological insight into clinically sensitive and insensitive genes. Although the shared functional annotations (cell communication, response to stimulus, signaling, and transport) by both gene sets are indicative of more reliable biological processes underlying ALFF alterations in MDD, the preferential enrichments of each gene set have special clinical relevance. Clinically sensitive genes were enriched for cell types of microglia and macrophage, developmental period from mid‐childhood to young adulthood, and biological processes of cell development and differentiation. The enriched time window of these genes is consistent with the common age window of the onset of MDD,^49^ suggesting a role of these genes in the onset of MDD. Due to microglia and macrophage are main immune cells in the brain, the cell type‐specific enrichment provides further support for the previously observed relation between immune and MDD.^50^, ^51^, ^52^, ^53^ The involvement of the immune mechanism in the current antidepressant treatments is supported by the observation that ketamine can program human monocytes into M2c‐like macrophages.^54^ Clinically insensitive genes were preferentially enriched for neurons, prenatal period, and synaptic signaling. The enrichment for neuron and synaptic signaling is consistent with the concept that ALFF is an indicator of regional spontaneous activity of neurons^6^, ^55^ and the role of synaptic dysfunction in MDD pathology.^56^, ^57^ The enrichment for prenatal period suggests that the genetically determined early neuronal and synaptic abnormalities may partially contribute to ALFF alterations in adult patients with MDD.^58^

Different from the significant genetic correlations of MDD with SCZ, ADHD, and BD,^59^ we only found significant overlap between genes associated with ALFF alterations in MDD and those associated with ALFF alterations in SCZ, which is consistent with the previous observation that MDD showed stronger transcriptome correlation in the cerebral cortex with SCZ than those with other psychiatric disorders.^36^ Moreover, clinically insensitive genes (66.82%) were more likely to be associated with ALFF alterations in SCZ than clinically sensitive genes (15.17%), which may reflect the similarity in molecular neuropathology. For instance, clinically insensitive genes were enriched for early developmental period, neuron, and synaptic signaling, which is in line with a neurodevelopmental model of SCZ characterized by neuronal and synaptic dysfunction.^60^, ^61^

Several limitations should be considered when interpreting our findings. First, the sample sizes of the two datasets are not large enough to generate very powerful results about ALFF differences between patients with MDD and healthy controls. Second, the transcription‐neuroimaging association results may be affected by some confounding factors, such as the differences in age, sex, and ancestry between gene expression data and neuroimaging data.^62^, ^63^, ^64^ Finally, the transcription‐neuroimaging association analysis was not conducted based on gene expression and neuroimaging data from the same individuals. The gene expression data were derived from six post‐mortem brains, whereas the neuroimaging data were derived from other living human brains. This approach was conducted based on the observation that the expression patterns of a portion of genes across brain structures are conserved between individuals.^35^ Therefore, our analyses can only identify ALFF‐alteration‐related genes with conserved across brain region expression patterns between individuals, which may miss those genes with great individual differences.

## 5 CONCLUSION

In this study, by conducting transcriptome‐neuroimaging association analyses in two independent neuroimaging datasets of MDD patients with different clinical features, we categorized genes associated with ALFF alterations in MDD into clinically sensitive and insensitive genes. These two sets of genes were differentially enriched for cortical differential expression genes in MDD, biological functions, developmental periods, cell types, and genes associated with ALFF alterations in SCZ. The results link macroscopic brain functional alterations in MDD and clinical heterogeneity to specific microscopic molecular events, which may shed light on the molecular mechanisms underlying ALFF alterations in MDD and inform novel targets for antidepressant treatments.

## AUTHOR CONTRIBUTIONS

Wenshuang Zhu and Chunshui Yu designed research. Wenshuang Zhu, Kaizhong Xue, and Yong Zhang acquired data. Wenshuang Zhu, Jilian Fu, Jie Tang and Kaizhong Xue analyzed data. Jilian Fu, Wen Qin, Feng Liu, and Chunshui Yu provided guidance and support. Wenshuang Zhu, Feng Liu and Chunshui Yu wrote the paper.

## CONFLICT OF INTEREST STATEMENT

The authors declare no conflict of interest.

## DATA AND CODE AVAILABILITY

Gene expression profiles are available in the Allen Human Brain Atlas (https://human.brainmap.org/static/download). Differential gene expression data of post‐mortem cerebral cortex are from Gandal et al. (https://www.science.org/doi/suppl/10.1126/science.aad6469/suppl_file/aad6469_gandal_sm_data‐table‐s1.xlsx). Temporal‐specific expression data were from cell‐specific expression analysis tool (http://genetics.wustl.edu/jdlab/csea‐tool‐2) which originally derived from the BrainSpan database (http://www.brainspan.org). Cell‐specific gene expression data can be downloaded from the CellMarker database (http://xteam.xbio.top/CellMarker/). The fMRI and T1‐weighted neuroimages of SCZ, BD, and ADHD are available in the OpenfMRI database (https://openfmri.org/dataset/ds000030/).

MRI data preprocessing and ALFF calculation were performed using DPARSFA version 4.3 (http://rfmri.org/DPARSF). The code for surrogate brain maps is available at https://github.com/murraylab/brainsmash. The code for human brain gene expression analysis can be found at https://github.com/BMHLab/AHBAprocessing. Enrichment for biological functions was performed in the online tool *g:Profiler* (https://biit.cs.ut.ee/gprofiler/). The code for ensemble‐based null model is available at https://github.com/benfulcher/GeneCategoryEnrichmentAnalysis.

## Supporting information


Figure S1.
Figure S2.

Table S1.
Table S2.
Table S3.
Table S4.
Table S5.
Table S6.
Table S7.
Table S8.
Table S9.
Table S10.

## Data Availability

The data that support the findings of this study are available in the supplementary material of this article.
