# Supplementary material for: Genes associated with spontaneous brain activity changes in clinically different patients with major depressive disorder: A transcription‐neuroimaging association study
Source: CNS Neurosci Ther. 2023 Jun 13;29(12):3913–24. doi: 10.1111/cns.14311 (PMC10651976; doi:10.1111/cns.14311)
Supplement: Supplementary file 1 — Figure S1. Figure S2. [file CNS-29-3913-s002.docx]

**Supplementary Materials**


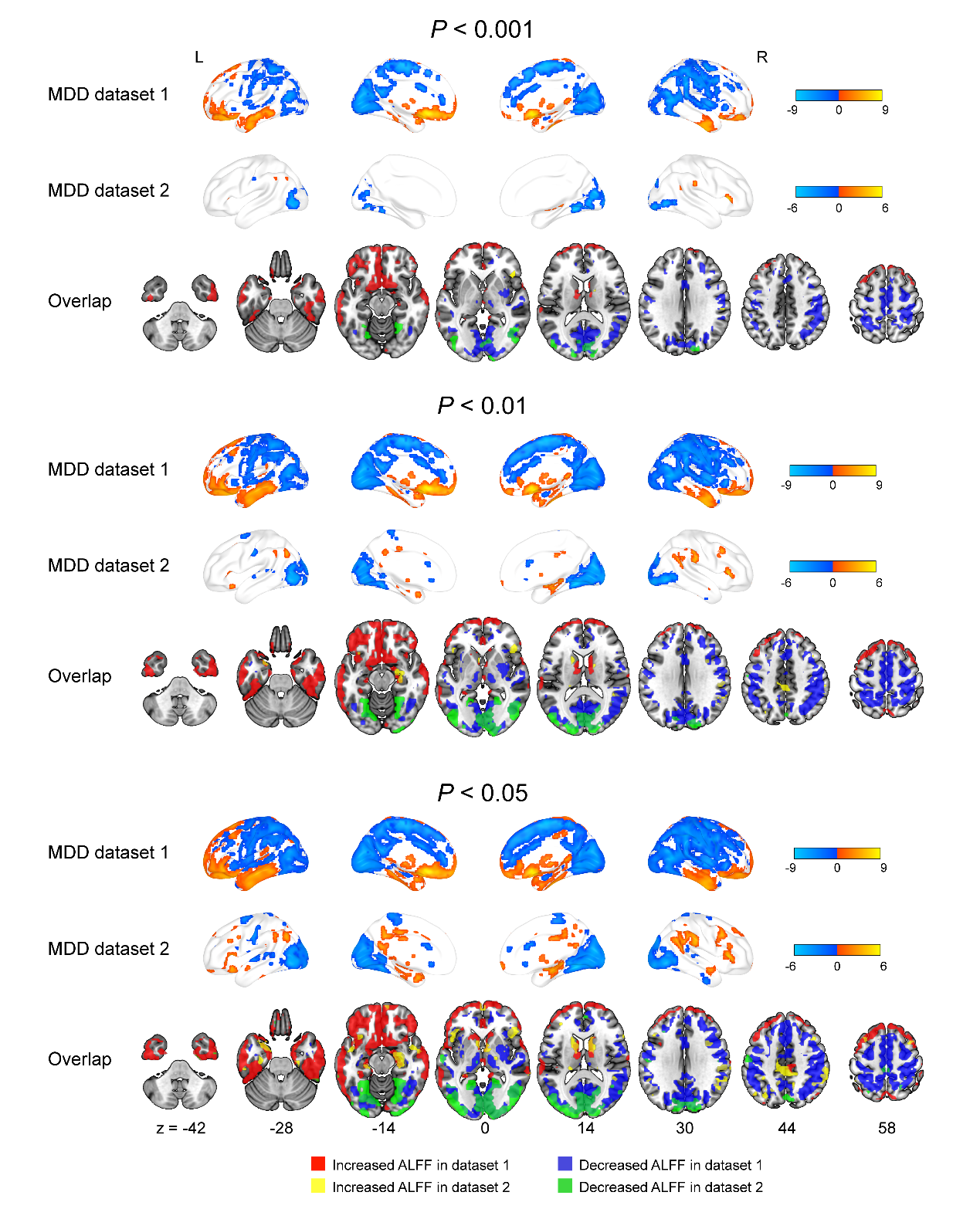


**Fig. S1. ALFF differences between patients with MDD and healthy controls and overlaps between the two datasets at different statistical thresholds.**


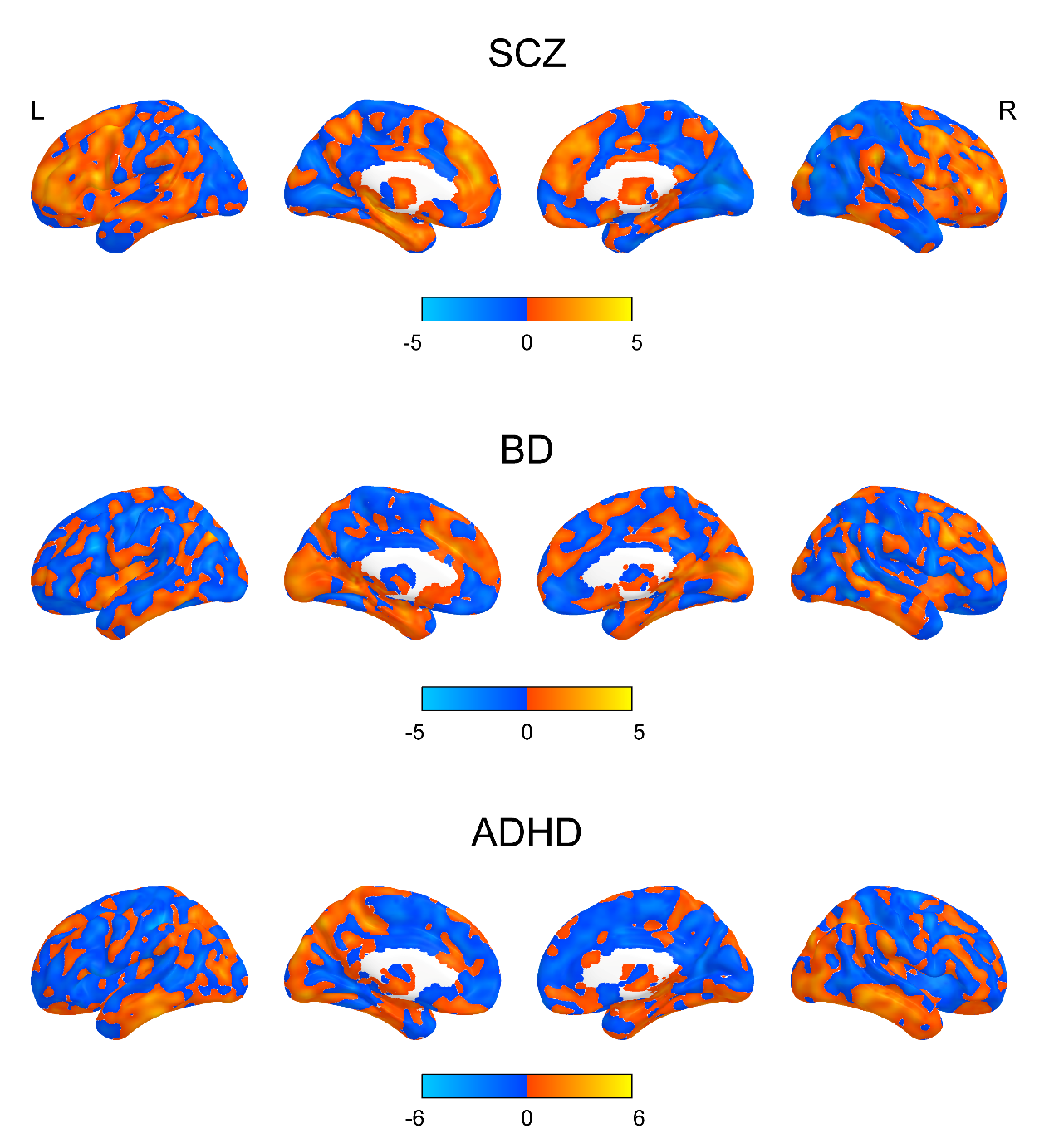


**Fig. S2. ALFF alterations in SCZ, BD and ADHD.**

The uncorrected *t*-maps of case-control ALFF alterations in SCZ, BD and ADHD. No significant differences were found when using the same multiple correction method as MDD (voxel-level FWE *P* < 0.05).
